# Predicting neurodevelopmental delay among two-year-old infants in Northern Uganda

**DOI:** 10.1080/16549716.2026.2748101

**Published:** 2026-10-15

**Authors:** David Mukunya, Grace Ndeezi, Gertrude Namazzi, Thorkild Tylleskär, Brendah Nambozo, Milton W. Musaba, Beatrice Odongkara, Vincentina Achora, Justin Bruno Tongun, Josephine Tumuhamye, Agnes Napyo, Vicent Ssentumbwe, Victoria Nankabirwa, James K. Tumwine

**Affiliations:** a Department of Community and Public Health, Busitema University, Mbale, Uganda; b Department of Research, Nikao Medical Center, Kampala, Uganda; c Department of Paediatrics and Child Health, School of Medicine, Makerere University College of Health Sciences, Kampala, Uganda; d Department of Health Policy, Planning and Management, School of Public Health, College of Health Sciences, Makerere University, Kampala, Uganda; e Center for Internation Health, Department of Global Public Health and Primary Care, University of Bergen, Bergen, Norway; f Department of Obstetrics and Gynaecology, Busitema University Faculty of Health Sciences, Mbale, Uganda; g Department of Paediatrics and Child Health, Gulu University, Gulu, Uganda; h Department of Obstetrics and Gynaecology, Gulu University, Gulu, Uganda; i Department of Paediatrics and Child Health, University of Juba, Juba, South Sudan; j Department of Research, Makerere University Hospital, Kampala, Uganda; k Department of Nursing, School of Medicine, Kabale University, Kabale, Uganda; l Division of Computational and Data Science, Washington University in St. Louis, St. Louis, MO, USA; m Centre for Intervention Science and Maternal Child Health (CISMAC), Centre for International Health, University of Bergen, Bergen, Norway; n Department of Epidemiology and Biostatistics, School of Public Health, Makerere University College of Health Sciences, Kampala, Uganda; o Department of Paediatrics and Child Health, Kabale University, Kabale, Uganda

**Keywords:** Early child development; thriving, human capacity development, low-income countries, neurodevelopmental delay, human capital, risk stratification

## Abstract

**Background:**

Data on neurodevelopmental disabilities in sub-Saharan Africa are scarce. Identifying children at the highest risk of neurodevelopmental delay could enable health workers to target these groups more effectively, improve resource allocation and programmatic focus.

**Objective:**

To develop and assess a model for predicting neurodevelopmental delay among two-year-old infants in Northern Uganda using easily obtainable predictors.

**Methods:**

We studied children born to women enrolled in a cluster randomized controlled trial (January 2018-March 2019) evaluating the effect of peer counseling on health facility births. At the trial’s final visit (day 50), 1,804 children were enrolled. In November 2020, we followed up 1,421 children (78.8%) and assessed their neurodevelopment at 2 years using the Malawi Developmental Assessment Tool. We used least absolute shrinkage and selection operator penalized regression with 10-fold cross-validation to select the best subset of predictors and assessed model performance using the area under the curve (AUC).

**Results:**

Of the 1,421 children, 70 (4.9%; 95% CI: 3.6–6.7%) had neurodevelopmental delays. Of the 19 candidate predictors, six were retained in the final model (lambda = 0.0076): moderate wasting, prolonged neonatal hospitalization (up to 28 days), maternal education, place of birth, preterm birth, and absence of skin-to-skin contact at birth. The model showed strong discriminative power (AUC 0.75) and reasonable calibration (calibration slope 1.37; calibration belt 0.536–0.727).

**Conclusion:**

We developed a promising tool for identifying infants at increased risk of neurodevelopmental delay in low-resource settings, which could help prioritize children for follow-up and specialized interventions.

## Paper Context

**Main findings:** we developed and evaluated a risk prediction model that identified six readily available factors associated with neurodevelopmental delay among infants in Northern Uganda and demonstrated a good ability to distinguish children at increased risk.

**Added knowledge:** this study provides evidence that routinely collected maternal, birth, and early infant characteristics can be used to support early identification of children who may benefit from targeted neurodevelopmental follow-up.

**Global health impact for policy and action:** the findings provide a practical approach for low-resource settings to improve early detection, prioritize limited resources, and guide interventions to reduce the burden of neurodevelopmental disabilities.

## Background

Over 250 million infants in low- and middle-income countries (LMICs) do not fulfill their neurodevelopmental potential, which results in poor school performance and suboptimal life achievements [1,2]. The estimated economic costs are between 28 and 74 billion dollars annually [3]. Interventions to address neurodevelopmental delay (NDD) are most effective if delivered in the first 3 years of life [4]. Unfortunately, the majority of children in sub-Saharan Africa are identified when it is too late to intervene. Delay in diagnosis is partly due to the cumbersome and resource intense nature of diagnosing NDD. Specialists in psychology, neurology or childcare can only deliver most NDD batteries and these professionals are very few in most LMICs and when present, they are in urban areas.

Developing a risk prediction model is key in identifying children who are most vulnerable and at risk of NDD. The philosophy of risk stratification has been encouraged by the World Health Organization Risk Stratification Working Group [5], which states that identifying children at the highest risk of NDD, especially in resource-constrained settings, will enable health workers to target these groups more effectively and improve resource allocation and programmatic focus. In addition, identifying children at lower risk of NDD will enable health workers to redirect limited resources to achieve maximal impact [6].

There is a paucity of data on the risk factors and models for neurodevelopmental disabilities in sub-Saharan Africa [7]. The majority of models on NDD are from high ‑income countries, but they are still limited in number and functionality for community ‑based settings [8]. However, given the unique nature of risk factors for NDD in LMICs such as malnutrition, infections and birth complications, models developed in high ‑income countries may not apply to LMICs. Moreover, if a model is going to be useful in community settings in sub-Saharan Africa, it should use predictors that are commonly recorded and easily obtainable in such settings [8]. This study aimed to develop a prediction model for NDD among children in LMICs to guide targeted screening and intervention in resource-limited settings.

## Methods

### Study design

This was a prospective cohort that followed up children from the SURVIVAL PLUSS cohort, which was conducted, between January 2018 and March 2019. The trial was registered in ClinicalTrials.gov, and the trial registration number is NCT02605369, registration date 2015–11–12.

### Setting

Our study was among infants born to women enrolled in a cluster randomized controlled trial evaluating the effect of peer counselling on health facility births (SURVIVAL PLUSS STUDY registered on ClinicalTrials.gov as NCT02605369) [9] in Lira district in northern Uganda. Lira district is approximately 340 km from the capital city, Kampala, and has 13 sub-counties, 1 municipality, and 751 villages. We recruited children from the SURVIVAL PLUSS cohort from Aromo, Agweng and Ogur sub ‑counties located in the northern part of the district. These sub ‑counties were chosen because of poor maternal and childhood indices. At the time of the study, the population of Lira district was ~400,000 people [9].

### Parent cohort

The parent cohort was a community-based cluster randomized controlled trial designed to evaluate the effect of a combined intervention on the proportion of mothers giving birth in health facilities. The combined intervention consisted of peer counseling, mobile phone messaging and distribution of mama kits. The unit of randomization was a cluster, made up of 5 to 10 villages with a population of >1,000 people. Up to 30 clusters were randomized (ratio of 1:1) to the intervention or control arm. Mothers were enrolled in the third trimester of pregnancy and followed up for 50 days postpartum. Each village had a recruiter (pregnancy monitor) who was elected during a community meeting, and who notified the research team of all pregnant women in her village during the study period, and of all births. During home visits, research assistants recruited women of 28 or more weeks pregnant who were resident in the selected clusters. They were followed up on days 1, 7, 28 and 52 postpartum [9]. On the last visit in the trial, day 50 after birth, there were 1804 children in the trial. In November and December 2020, we tracked 1421 children (78.8%) and assessed their neurodevelopment; their mean age was 2.4 years and the standard deviation was 0.23 years.

### Participants: inclusion and exclusion

We included children aged between 1.8 years and 3.8 years, born to women who participated in a cluster randomized controlled trial in Northern Uganda.

### Sample size

This is a post hoc secondary analysis of children followed up at 2 years in the SURVIVAL Plus trial, which was powered for its primary outcome, facility-based birth. The sample size for the present analysis was therefore fixed by the 1,421 children with complete two-year neurodevelopmental assessments, and all were included. We assessed its adequacy for developing a prediction model using the criteria of Riley *et al*. [10]. Assuming a prevalence of neurodevelopmental delay of [3.2%] and a Cox-Snell R^2^ of 0.2, 18 candidate predictor parameters would require at least 1,299 participants.

### Study procedures

Mothers or caregivers of infants enrolled in the trial were contacted by telephone using the locator details captured on locator forms at baseline/enrolment. They were invited to bring their children to Lira Regional Referral Hospital for neurodevelopmental assessment. Trained research midwives obtained written informed consent from the mothers/caregivers and then collected data on the child’s nutrition and medical history.

Anthropometric measurements were taken by the research assistants, including weight, measured in duplicate to the nearest 100 g using electronic scales (Seca model 8811021659, Hamburg, Germany), and length, measured in duplicate to the nearest 1 mm using a wooden length board. The Stata package ‘zscore06’ was used to calculate weight-for-height z-score (WHZ) and height-for-age z-score (HAZ). Mid-upper arm circumference was measured in duplicate to the nearest 1 mm at the midpoint between the olecranon and acromion process of the left arm, using a standard measuring tape. Overall inter-rater reliability was substantial, with a kappa value greater than 0.75.

A food diversity questionnaire was administered, with minimum acceptable diet defined as consumption of at least four of seven food groups and a minimum of four meals per day. Foods were grouped into seven categories: roots and tubers, legumes, grains, dairy products, eggs, flesh foods, vitamin A-rich fruits and vegetables; and other fruits and vegetables [10]. Children who met the minimum acceptable diet criteria were coded as 1, and those who did not were coded as 0.

Neurological assessments were conducted by trained research assistants using the Malawi Developmental Assessment Tool (MDAT) to evaluate each child’s neurodevelopment. Items were scored as ‘pass’ or ‘fail,’ with ‘don’t know’ recorded when a child was uncooperative. The language and fine motor domains capture cognitive skills such as problem-solving, attention, and early numeracy (for example, asking the child to find an object hidden under a cloth). When a child failed six consecutive items in a domain, the assessor moved on to the next domain [11]. MDAT assessments were carried out by two trained research midwives. These were trained by a team comprised of a pediatric neurologist and two pediatricians who were experienced in neurodevelopmental evaluation and another pediatrician who had conducted neurodevelopment assessments in the Iganga-Mayuge DSS as a part of her PhD [11]

### Study measures

#### Neurodevelopmental delay

We used the MDAT to measure neurodevelopment delay [12]. MDAT is a validated tool that has been used in other resource limited settings to measure neurodevelopment in children from birth to 6 years in four domains: gross motor, fine motor, and language development through direct observation of the child and social development by interviewing the caregiver.

#### Candidate predictors

These included maternal characteristics such as maternal age, wealth index, maternal education, parity, place of birth, complications during childbirth and postpartum depression. We also included child characteristics like; gestational age at birth, skin ‑to ‑skin contact at birth, hypothermia, hypoglycemia, wasting, exclusive breastfeeding, initiation of breastfeeding, sex of the child, prolonged neonatal hospitalization (up to 28 days), history of severe childhood illness, resuscitation at birth and history of convulsions.

### Data analysis

Data were analyzed using Stata version 19.0 (StataCorp; College Station, TX, USA). Continuous variables were summarized into means, medians, and their measures of dispersion. Categorical variables were summarized as proportions. Analysis of the risk of neurodevelopmental delay factored in the clustered nature of the data in variance estimation.

#### Model development

We used least absolute shrinkage and selection operator (LASSO) logistic regression to select predictors that contributed most to predicting NDD. Candidate neonatal, maternal, care-related, and socioeconomic variables were specified as a priori based on prior evidence [12–15]. LASSO performs data shrinkage by applying a penalty factor (λ) to the regression coefficients. The best penalty factor (λ) is then selected using 10-fold cross-validation with bootstrapping [16]. Applying the penalty results in shrinking the small coefficients to zero, hence eliminating the least contributing variables from the model. The penalization also ensures that very large coefficients are shrunk to lesser values. This way, penalization avoids extreme predictions.

Predicted probabilities of NDD were generated from the final penalized model. Model calibration was assessed by plotting predicted probabilities against observed outcomes and estimating the calibration slope. Model discrimination was evaluated using the cross-validated area under the receiver operating characteristic curve (AUC), obtained through 10-fold cross-validation with a separate random seed to reduce optimism bias. A smoothed lowess curve was used to visualize discrimination performance across the range of predicted risks.

## Results

The parent study enrolled 1887 pregnant women. One died during pregnancy. Of the remaining 1886 women, 1804 were visited on Day 50, which was the last mandatory visit of the parent study. Of these, we traced and evaluated 1421 children (78.8%) for neurodevelopment at approximately 2 years of age (Figure 1). Of the 1421 children, 70 (4.9%: 95% CI [3.6%, 6.7%]) had neurodevelopmental delays.

**Figure 1.**
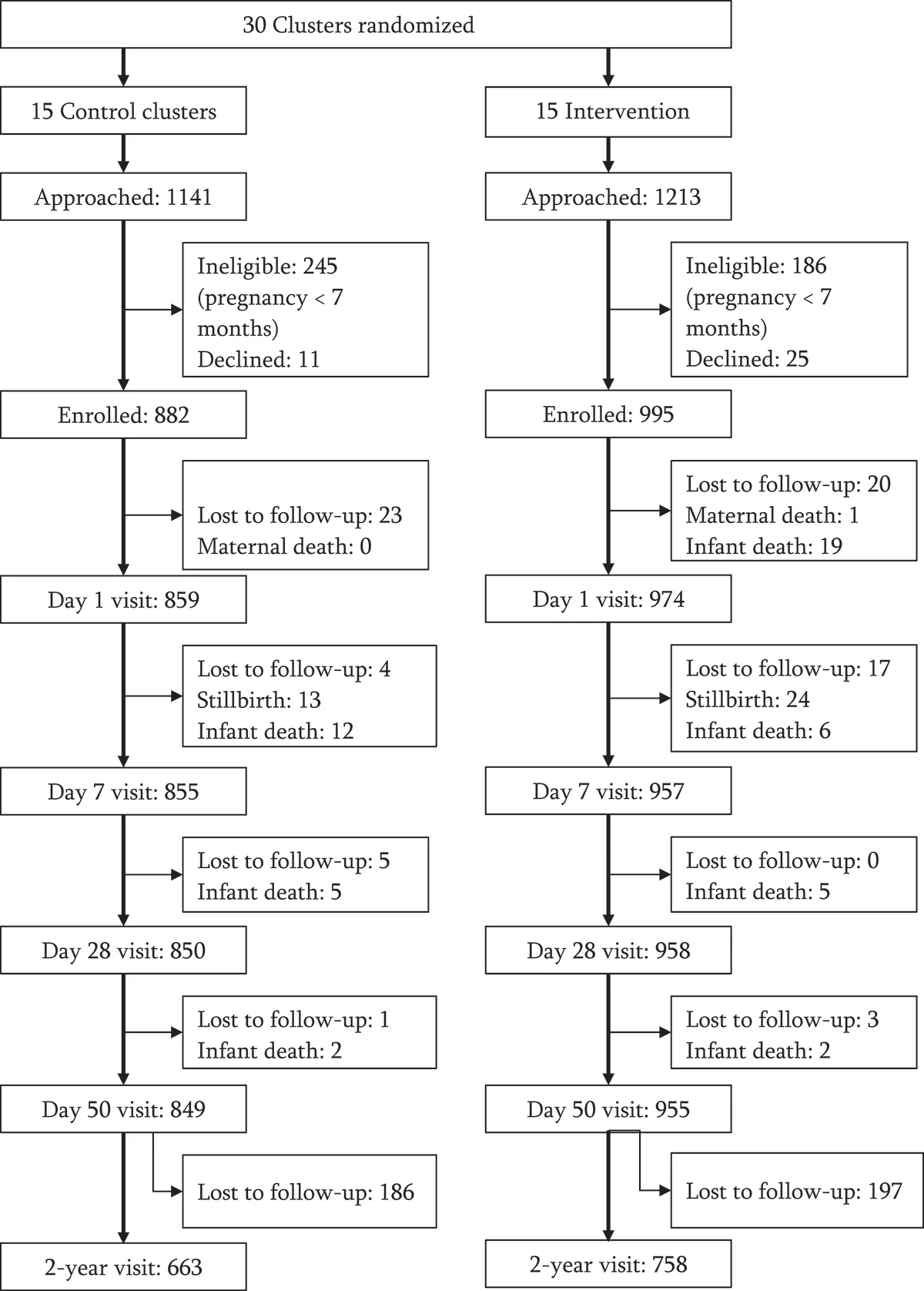
Study profile of 2-year-old infants in Northern Uganda evaluated for neurodevelopment.

### Participants characteristics

The median maternal age was 23 years, IQR (19–29) at baseline. Fifty-six percent (798/1421) of mothers were aged 24 years and below, 44% (624/1421) were poor, and the majority 86.5% (1229/1421) had attained primary or higher level of education. Sixty ‑seven percent (957/1421) of the infants were born at health facilities, half 51.1% (726/1421) of the infants were female, and 13.3% (189/1421) were born before 37 weeks of gestation (Tables 1 and 2).

**Table 1.** Characteristics of women that participated in the SURVIVAL-PLUS trial in northern Uganda that were assessed for neurodevelopment.

Characteristic	Neurodevelopmental delay		
	No n (%)	Yes n (%)	Total n (%)
N	1,351 (95.1)	70 (4.9)	1,421 (100.0)
Maternal age			
1. ≤ 24	762 (56.4)	36 (51.4)	798 (56.2)
2. 25–35	476 (35.2)	20 (28.6)	496 (34.9)
3. > 35	113 (8.4)	14 (20.0)	127 (8.9)
Wealth index			
Poor	585 (43.3)	39 (55.7)	624 (43.9)
Medium	241 (17.8)	13 (18.6)	254 (17.9)
Rich	525 (38.9)	18 (25.7)	543 (38.2)
Maternal education			
None	174 (12.9)	18 (25.7)	192 (13.5)
Primary or more	1,177 (87.1)	52 (74.3)	1,229 (86.5)
Parity			
Nulliparity	753 (55.7)	38 (54.3)	791 (55.7)
Multiparity	598 (44.3)	32 (45.7)	630 (44.3)
Intrapartum complication (maternal)			
No	678 (50.2)	37 (52.9)	715 (50.3)
Yes	673 (49.8)	33 (47.1)	706 (49.7)
Place of birth			
Home	431 (31.9)	33 (47.1)	464 (32.7)
Health facility	920 (68.1)	37 (52.9)	957 (67.3)
Postpartum depression			
No depression	832 (61.9)	46 (65.7)	878 (62.1)
Depression	512 (38.1)	24 (34.3)	536 (37.9)
Preterm 37			
<37 weeks	1,176 (87.0)	56 (80.0)	1,232 (86.7)
37 or more weeks	175 (13.0)	14 (20.0)	189 (13.3)
Intervention status			
Control	624 (46.2)	39 (55.7)	663 (46.7)
Intervention	727 (53.8)	31 (44.3)	758 (53.3)

**Table 2.** Characteristics of infants born to women that participated in the SURVIVAL-PLUS trial in northern Uganda that were assessed for neurodevelopment.

Characteristic	Neurodevelopmental delay		
	No n (%)	Yes n (%)	Total n (%)
**N**	**1,351 (95.1)**	**70 (4.9)**	**1,421 (100.0)**
Sex of the child			
Male	662 (49.0)	33 (47.1)	695 (48.9)
Female	689 (51.0)	37 (52.9)	726 (51.1)
Birth asphyxia			
No	926 (81.5)	52 (86.7)	978 (81.8)
Yes	210 (18.5)	8 (13.3)	218 (18.2)
Initiation of breastfeeding			
Early	850 (63.1)	40 (57.1)	890 (62.8)
Late	497 (36.9)	30 (42.9)	527 (37.2)
Exclusive breastfeeding			
No	295 (21.9)	17 (24.3)	312 (22.0)
Yes	1,050 (78.1)	53 (75.7)	1,103 (78.0)
History of child hospitalization			
Yes	953 (83.9)	51 (85.0)	1,004 (83.9)
No	183 (16.1)	9 (15.0)	192 (16.1)
Prolonged neonatal hospitalization (up to 28 days)			
No	1,109 (97.6)	55 (91.7)	1,164 (97.3)
Yes	27 (2.4)	5 (8.3)	32 (2.7)
Skin-to-skin contact at birth			
No	273 (20.2)	19 (27.1)	292 (20.5)
Yes	1,078 (79.8)	51 (72.9)	1,129 (79.5)
Hypothermia			
No	814 (60.3)	37 (52.9)	851 (59.9)
Yes	537 (39.7)	33 (47.1)	570 (40.1)
Hypoglycemia			
No	1,326 (98.1)	67 (95.7)	1,393 (98.0)
Yes	25 (1.9)	3 (4.3)	28 (2.0)
Convulsions			
No	1,339 (99.1)	70 (100.0)	1,409 (99.2)
Yes	12 (0.9)	0 (0.0)	12 (0.8)
Moderate wasting (child) at NDD assessment			
No	1,318 (98.7)	58 (89.2)	1,376 (98.3)
Yes	17 (1.3)	7 (10.8)	24 (1.7)

### Predictors of neurodevelopmental delay among infants

#### Model development

Initially, 19 candidate predictors were included in developing the model. These included maternal age, wealth index, maternal education, parity, place of birth, complications during childbirth, place of birth, postpartum depression, gestational age at birth, skin -to -skin contact at birth, hypothermia, hypoglycemia, moderate wasting, exclusive breast feeding, initiation of breastfeeding, history of severe childhood illness, sex of the child, prolonged neonatal hospitalization (up to 28 days) after birth, resuscitation at birth and history of convulsions. After performing LASSO logistic regression with 10-fold cross-validation, only six predictors: moderate wasting, prolonged neonatal hospitalization (up to 28 days), maternal education, place of birth, preterm birth (before 37 weeks of gestation) and no skin-to-skin contact at birth were retained in the final model using a lambda value of 0.0076 (Table 3). Crude and adjusted odds ratios for all 19 candidate predictors, including those not retained by LASSO, are presented in Table S1.

**Table 3.** Lasso regression for the prediction of neurodevelopmental delay among infants.

Predictor variable	Penalized coefficients
Moderate wasting at NDD assessment	−2.24
Neonatal hospitalization up to 28 days	−1.05
No maternal education	0.56
Home birth	0.51
Preterm birth (<37 weeks)	−0.18
Skin-to-skin contact at birth	0.04

#### Model performance

The final risk prediction model demonstrated strong discriminative power to differentiate the infants with neurodevelopmental delay and those without, AUC 0.75 (Figure 2) and a decent calibration with a calibration slope of 1.37 and a fair calibration belt of 0.536–0.727 (Figure 3 and Table 4).

**Figure 2.**
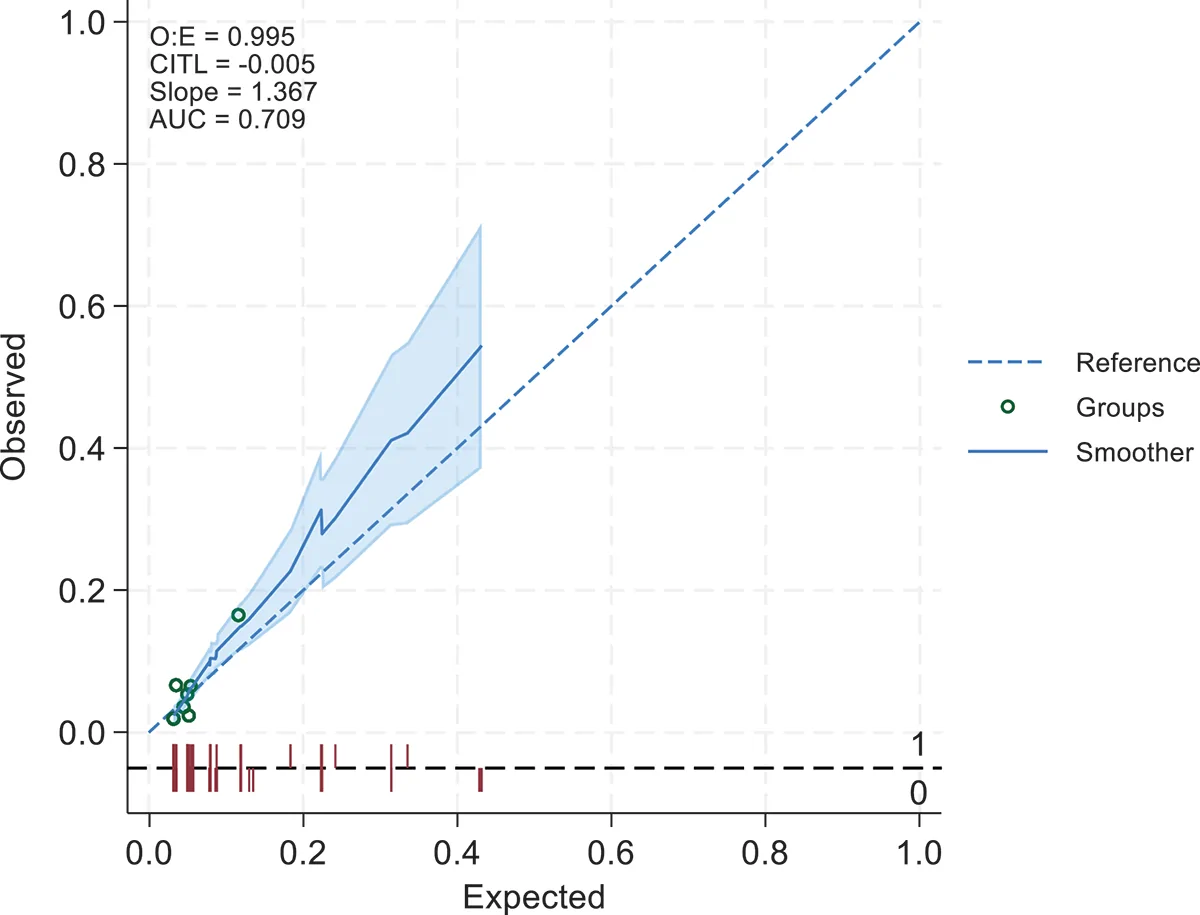
A calibration plot for the performance of the risk prediction model for neurodevelopmental delay among infants.

**Figure 3.**
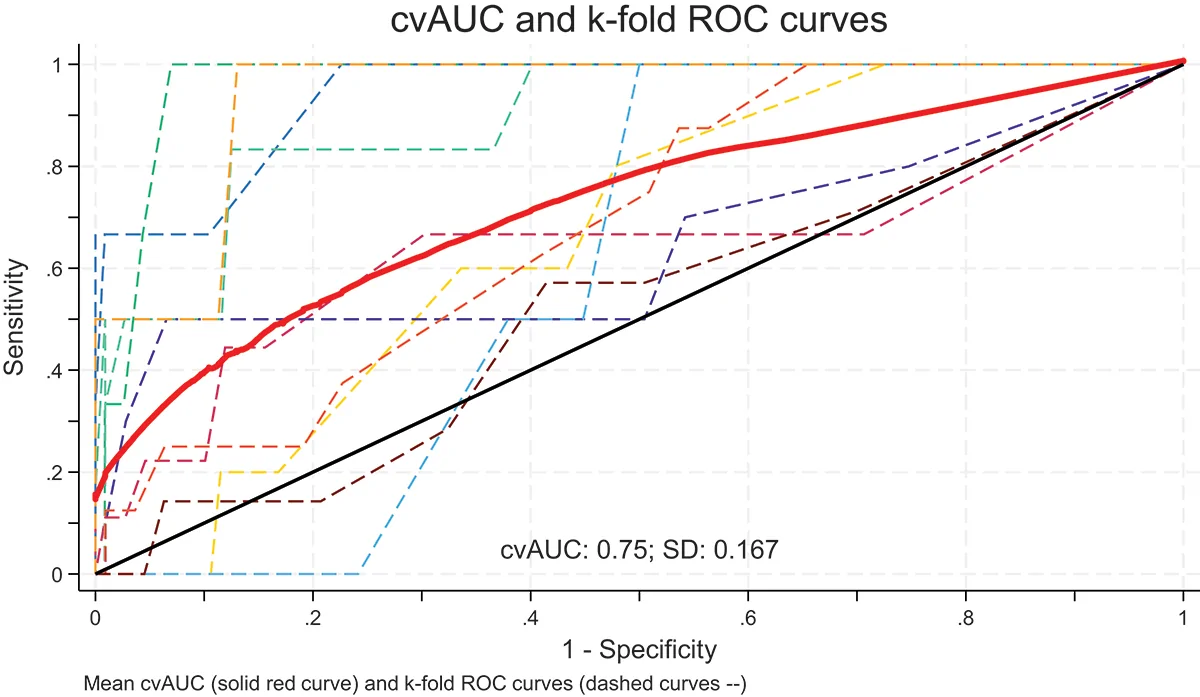
Receiver operating characteristic curve for a model predicting neurodevelopmental delay among infants.

**Table 4.** Discrimination performance of the lasso prediction model for neurodevelopmental delay.

Performance metric	Estimate
Cross validated AUC	0.74
Standard deviation across folds	0.17
95 CI for AUC	0.536–0.727
Number of folds	10
Validation approach	10 -fold cross validation

### Individualized risk prediction

The model was used to calculate the risk for NDD for a hypothetical participant with the following characteristics. The participant had moderate wasting (coded as 1), prolonged neonatal hospitalization (up to 28 days) (coded as 1), no maternal education (coded as 0), residing in a rural area (coded as 0), had a preterm birth of <37 weeks (coded as 1), and did not receive skin ‑to ‑skin contact at birth (coded as 0). In other words, this participant represents the reference category for all six predictors retained in the final model. The risk score for this participant is calculated in the equation below:

Risk Score =  1 / (1 + exp (2.669* moderate wasting) + (1.539*hospitalization) + (−0.813 × maternal education) + (−0.751* place of birth) + (0.520 × preterm birth) + (−0.219 × skin ‑to ‑skin contact at birth). Thus, the individualized risk of NDD for this hypothetical participant derived from the model is 97.58%.

## Discussion

We developed a model that predicts neurodevelopmental delay among infants using maternal sociodemographic and perinatal characteristics. Our findings are consistent with existing evidence and suggest that child intrinsic/biological and caregiver/maternal vulnerabilities can negatively impact the neurodevelopment of children. We did not find a model predicting neurodevelopmental delay in sub-Saharan Africa; therefore, we compared our model to models from high ‑income settings. Our model performance was similar to one predicting neurodevelopmental delay among preterm babies in Taiwan, which found an AUC between 0.75 and 0.78 (95% CI, 0.72–0.82) for cognitive delay with 10 variables and an AUC of 0.73–0.82 (95% CI, 0.72–0.85) for motor delay with 4 variables [17]. Our model is also similar to another model from China that predicted Autism, Global Language Delay and Developmental Language Delay with an AUC of 78% [18]. A model developed in the USA performed much better than our model with an AUC of 84% [8]. The difference could be explained by the fact that the model predicted delay at 4 years of age and included markers of development, such as the ability to smile and speak [8].

These findings highlight the need for interventions that address not only the clinical factors, but also modifiable maternal socio-demographic characteristics that affect the neurodevelopment of infants. Our results have direct clinical implications for practice in low-resource settings. The model identifies specific subgroups of infants at highest risk for neurodevelopmental delay, enabling clinicians to prioritize the scarce resources and stratify them for close follow-up and early interventions or management. The predictors retained in the final model can be assessed with brief, standardized tools during routine maternal or child contact with the health system. For instance, maternal age, level of education, prematurity, skin ‑to ‑skin contact at birth and place of birth can be assessed during the intrapartum period or immediate postpartum for the women who give birth at home. Some predictors, such as neonatal hospitalization, can be assessed during the neonatal period, while moderate wasting could be assessed during routine immunisation visits, as these typically include growth monitoring. Neurodevelopmental delay could be assessed during the routine immunisation visits using age-appropriate validated tools. There is a need to develop a tool or calculator for use by healthcare practitioners, since implementing it would require minimal infrastructural support the model relies on non-laboratory variables that are feasible to collect in low-resource settings. Furthermore, these findings highlight the need for targeted follow-up and enhanced care for infants with moderate wasting, preterm birth, or prolonged neonatal hospitalization, as these factors markedly increase vulnerability in the first months of life. Strengthening facility-based delivery and ensuring consistent implementation of kangaroo mother care can mitigate some of the identified risks. Integrating these predictors into routine newborn and pediatric assessments may help clinicians more accurately identify high-risk infants and prioritize early interventions that support survival, growth, and long-term development. However, differences in health-system capacity, access to neonatal and developmental services, and socioeconomic conditions may affect the model’s performance. External validation in other populations is therefore needed before wider application.

The main strength of the current model is the use of a large sample size, recruited from a rural setting in northern Uganda. Potentially, our outcome is generalizable to similar populations (socio-economic and health-care constraints) in low-resource settings. In addition, we employed a LASSO regression model in selecting the predictors (which can handle multicollinearity, minimize overfitting, and shrink less important predictors to zero, producing a more parsimonious model), and bootstrapping. Our study is among the first studies in SSA to develop a risk prediction model for neurodevelopmental delay among infants. Nonetheless, our study only assessed developmental delay at 2 years of age, therefore, some developmental difficulties that emerge at older ages were not captured. Longitudinal follow-up with repeated developmental assessments at later ages would be valuable for evaluating developmental trajectories and the longer-term predictive performance of the model.

## Conclusion

Our model is a good tool for identifying infants at an increased risk of neurodevelopmental delay in low ‑resource settings. Developing such models and tools could be an important step to stratify infants at risk of neurodevelopmental delay right from birth. This would ensure timely screening and administration of interventions for infants at the greatest risk of neurodevelopmental delay. Researchers could use this model as a foundation for refining future models to achieve better precision.

## Supplementary Material

Supplementaryfile.docx

STROBE checklist NDDSURV.docx

## Data Availability

The datasets used and/or analysed during the current study are available from the first author on reasonable request.
