# Supplementary material for: Predicting neurodevelopmental delay among two-year-old infants in Northern Uganda
Source: Glob Health Action. 2026 Oct 15;19(1):2748101. doi: 10.1080/16549716.2026.2748101 (PMC13674651; doi:10.1080/16549716.2026.2748101)
Supplement: Supplementary file 2 [file ZGHA_A_2748101_SM3215.docx]

## **SUPPLEMENTARY FILE**

## **Table S1. Crude and adjusted odds ratios for all 19 candidate predictors of neurodevelopmental delay (NDD)**

| Predictor | Crude OR (95% CI) | p-value | Adjusted OR (95% CI)ᵇ | p-value | Retained by LASSO |
| --- | --- | --- | --- | --- | --- |
| Preterm birth ( Ref: <37 weeks) | 1.68 (0.92–3.08) | 0.094 | 1.74 (0.89–3.42) | 0.108 | Yes |
| Skin to skin contact at birth (ref: No) | 0.68 (0.39–1.17) | 0.164 | 0.80 (0.42–1.54) | 0.508 | Yes |
| Hypothermia | 1.35 (0.84–2.19) | 0.220 | 1.01 (0.57–1.81) | 0.961 | No |
| Hypoglycemia | 2.37 (0.70–8.06) | 0.165 | 1.64 (0.34–8.03) | 0.540 | No |
| Wealth index | 1.65 (1.02–2.67) | 0.043 | 1.29 (0.72–2.30) | 0.396 | No |
| Maternal education: (Ref: No education) | 0.43 (0.24–0.75) | 0.003 | 0.43 (0.21–0.87) | 0.020 | Yes |
| Moderate wasting | 9.36 (3.73–23.45) | <0.001 | 16.89 (5.32–53.63) | <0.001 | Yes |
| Maternal age | 0.82 (0.51–1.32) | 0.414 | 0.50 (0.19–1.31) | 0.158 | No |
| Exclusive breastfeeding | 0.88 (0.50–1.54) | 0.644 | 0.90 (0.45–1.81) | 0.766 | No |
| Delayed initiation of breastfeeding | 1.28 (0.79–2.09) | 0.316 | 1.23 (0.69–2.21) | 0.480 | No |
| Place of birth ( | 0.53 (0.32–0.85) | 0.009 | 0.48 (0.26–0.87) | 0.015 | Yes |
| Child sex | 1.08 (0.67–1.74) | 0.762 | 1.07 (0.60–1.89) | 0.828 | No |
| Postpartum depression | 0.85 (0.51–1.41) | 0.522 | 0.75 (0.40–1.38) | 0.348 | No |
| Parity | 1.06 (0.65–1.72) | 0.812 | 2.22 (0.84–5.85) | 0.108 | No |
| Maternal complication (ref: No) | 0.90 (0.56–1.45) | 0.663 | 1.20 (0.67–2.17) | 0.538 | No |
| Neonatal hospitalization up to day 28 (ref: No) | 3.73 (1.38–10.07) | 0.009 | 5.27 (1.77–15.67) | 0.003 | Yes |
| Severe illness (ref: Yes) | 0.92 (0.44–1.90) | 0.820 | 1.45 (0.67–3.15) | 0.349 | No |
| Birth asphyxia | 0.68 (0.32–1.45) | 0.317 | 0.70 (0.30–1.63) | 0.406 | No |
| History of convulsions | Not estimableᵃ | — | Not estimableᵃ | — | No |

*CI, confidence interval; OR, odds ratio.*

*ᵃ Odds ratio for convulsions could not be estimated because of perfect prediction: all cases with convulsions did not have the outcome (NDD), so the coefficient and its confidence interval are not identified.*

*ᵇ Adjusted odds ratios are from a single multivariable logistic regression model including all 19 candidate predictors simultaneously (unpenalized), fit to allow direct comparison with the crude (unadjusted) estimates.*
